# Pulmonary Hypertension in Rare Disease Overlap: Neurofibromatosis Type 1 Meets Sarcoidosis

**DOI:** 10.7759/cureus.98767

**Published:** 2025-12-08

**Authors:** Akshay Sharma, Yasmin Bilal, Imran Yousaf, Pardeep Masuta, Deepak Gupta

**Affiliations:** 1 Medicine, Luminis Health Anne Arundel Medical Center, Annapolis, USA; 2 Division of Rheumatology, Department of Medicine, TidalHealth, Salisbury, USA; 3 Division of Pulmonary and Critical Care, Department of Medicine, TidalHealth, Salisbury, USA

**Keywords:** breast cancer, leg edema, neurofibromatosis type 1 (nf1), pulmonary hypertension, pulmonary sarcoidosis

## Abstract

We present a case of pulmonary hypertension (PH) and right heart failure in a 66-year-old female patient with longstanding neurofibromatosis Type I (NF1) since 1973 and sarcoidosis since 2009. She had a history of breast cancer 30 years ago, which was treated with left mastectomy followed by chemotherapy, and she has remained in remission thereafter. We detail her clinical presentation, diagnostic evaluation, and therapeutic course, highlighting the diagnostic challenges posed by multifactorial PH and the implications for targeted management.

## Introduction

Pulmonary hypertension (PH) is a rare complication of neurofibromatosis Type 1 (NF1) and pulmonary sarcoidosis. NF1 is an autosomal dominant genetic disorder that primarily affects the nervous system, with an estimated prevalence of approximately 1 in 3,000-4,000 individuals [1]. NF1 is associated with a spectrum of arterial vasculopathies, including the rare comorbidity of pulmonary arterial hypertension (PAH), which has been documented in several case reports [2-4]. In recognition of its multifactorial pathogenesis, NF1 was classified under Group 5 PH at the 4th World Symposium on Pulmonary Hypertension in 2008 [5]. 

Sarcoidosis is a systemic granulomatous disease that predominantly involves pulmonary parenchyma, thoracic lymph nodes, airways, and vasculature [6]. Its prevalence varies significantly by geographic region, ranging from 1-5 cases per 100,000 in East Asia to 140-160 cases per 100,000 in Sweden and Canada. In the United States, the estimated prevalence is approximately 60 cases per 100,000 [6]. Sarcoidosis-associated PH (SAPH), a recognized complication, occurs in 3-20% of patients and may affect up to 74% of those with advanced disease. Like NF1-associated PAH, SAPH is also categorized under Group 5 PH due to its complex and multifactorial etiology [7]. 

We present an interesting case of severe PH in a postmenopausal woman with NF1 since 1973, and sarcoidosis diagnosed in 2009. She was diagnosed with breast cancer 30 years ago, which was treated with a left mastectomy followed by chemotherapy, and she has remained in remission since then. 

## Case presentation

**Figure 1:**
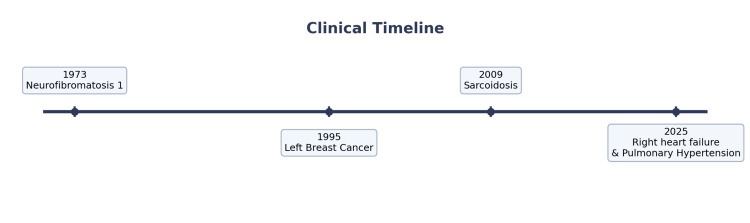
Clinical timeline of medical events

A 66-year-old female patient with sarcoidosis, NF1, asthma, and history of breast cancer presented with three weeks of progressively worsening exertional dyspnea (New York Heart Association (NYHA) class II at baseline), bilateral lower extremity swelling, and erythema with blistering ulceration on her lower legs [8]. She did not report orthopnea, cough, fever, or any recent travel. Her medical history included sarcoidosis since 2009 involving her lungs as confirmed by bronchoscopic biopsy, history of anterior uveitis and skin nodules. Her sarcoidosis was not currently active, and she was not on any immunosuppressive medications. She had also had NF1 since 1973. She had breast cancer in 1995, which was treated with left mastectomy and chemotherapy and has been in remission since then. She had mild intermittent asthma, hyperlipidemia, alopecia areata, and right bundle branch block. Her surgical history included removal of a cutaneous neurofibroma from her back in 2002 and her hand in 2006, left mastectomy, colectomy for a benign tumor, and spinal fusion. Prior to admission, her medications included an albuterol inhaler for asthma. She had a normal pulmonary function test (PFT) in 2014. A transthoracic echocardiogram (TTE) in 2019, demonstrated left ventricular ejection fraction (LVEF) of 60-65%, trace to mild mitral regurgitation, and trace tricuspid regurgitation. An exercise tolerance test done in July 2020 showed a Duke treadmill score of 3, indicating moderate risk. A Lexi scan Nuclear Stress Test in August 2020, was normal with ejection fraction (EF) 71% and without any regional wall motion abnormalities. Figure 1 shows the timeline of various medical events.

On presentation, she was tachycardic at 110 bpm. Physical exam showed decreased bilateral breath sounds, pitting edema of both legs, and erythematous blistering lesions over the anterior shins, more on the right side. Laboratory evaluation at admission were as shown in Table 1.

**Table 1:** Results of laboratory investigations Laboratory Investigations showed leukocytosis, AG acidosis, decreased renal functions, elevated liver enzymes, elevated NT-ProBNP suggestive of right heart failure, elevated CRP suggestive of inflammatory response and positive blood culture for MSSA consistent with sepsis. Hgb: Hemoglobin; WBC: White blood cell count; ESR: Erythrocyte sedimentation rate; AG: Anion gap; Cr: Creatinine; BUN: Blood urea nitrogen; AST: Aspartate aminotransferase; ALT: Alanine aminotransferase; T bil: Total bilirubin; D bil: Direct bilirubin; NT-ProBNP: N-terminal pro-B-type natriuretic peptide; TSH: Thyroid stimulating hormone; HbA1c: Hemoglobin A1c; CRP: C-reactive protein; RF: Rheumatoid factor; ANA: Antinuclear antibody; CCP: Cyclic citrullinated peptide; ACE: Angiotensin-converting enzyme; MSSA: Methicillin-sensitive *Staphylococcus auerus*

Test	Result	Reference
HEMATOLOGICAL		
Hgb	15.4 g/dL	12.0–15.5 g/dL
WBC	16.9 × 10³/µL (H)	4.0–10.0 × 10³/µL (female)
Platelets	220 × 10³/µL	150–400 × 10³/µL
ESR	4 mm/hour	0–30 mm/hour (female)
CHEMISTRY		
Sodium (Na)	139 mmol/L	135–145 mmol/L
Potassium (K^+^)	5.8 mmol/L (H)	3.5–5.1 mmol/L
Bicarbonate (HCO3^-^)	16 mmol/L (L)	22–29 mmol/L
Calcium (Ca_2_^+^)	10.8 mg/dL (corrected for albumin) (H)	8.6–10.2 mg/dL
Phosphorus	4.6 mg/dL (H)	2.5–4.5 mg/dL
AG	16 mmol/L (H)	8–12 mmol/L
Cr	1.46 mg/dL (H)	0.6–1.1 mg/dL
BUN	58.3 mg/dL (H)	7–20 mg/dL
AST	122 U/L (H)	10–40 U/L
ALT	154 U/L (H)	7–56 U/L
T bil	1.35 mg/dL (H)	0.2–1.2 mg/dL
D bil	0.72 mg/dL (H)	0.0–0.3 mg/dL
Albumin	3.3 g/dL (L)	3.5–5.0 g/dL
D-dimer	1.8 µg/mL FEU (H)	<0.5 µg/mL FEU
NT-ProBNP	6540 pg/mL (H)	<100 pg/mL
Troponin	20 ng/L (H)	<14 ng/L
TSH	2.85 µIU/mL	0.4–4.0 µIU/mL
HbA1c	6.3 % (H)	4.0–5.6 %
IMMUNOLOGY		
CRP	39 mg/L (subsequent: 41 → 28 mg/L) (H)	<5 mg/L
RF	Negative	Negative
ANA	Negative	Negative
Anti-CCP	Negative	Negative
ACE	47 U/L	8–52 U/L
MICROBIOLOGICAL		
Blood Culture	Positive for MSSA (Abnormal)	Negative
Hepatitis panel (A, B, C)	Negative	Negative

**Figure 2:**
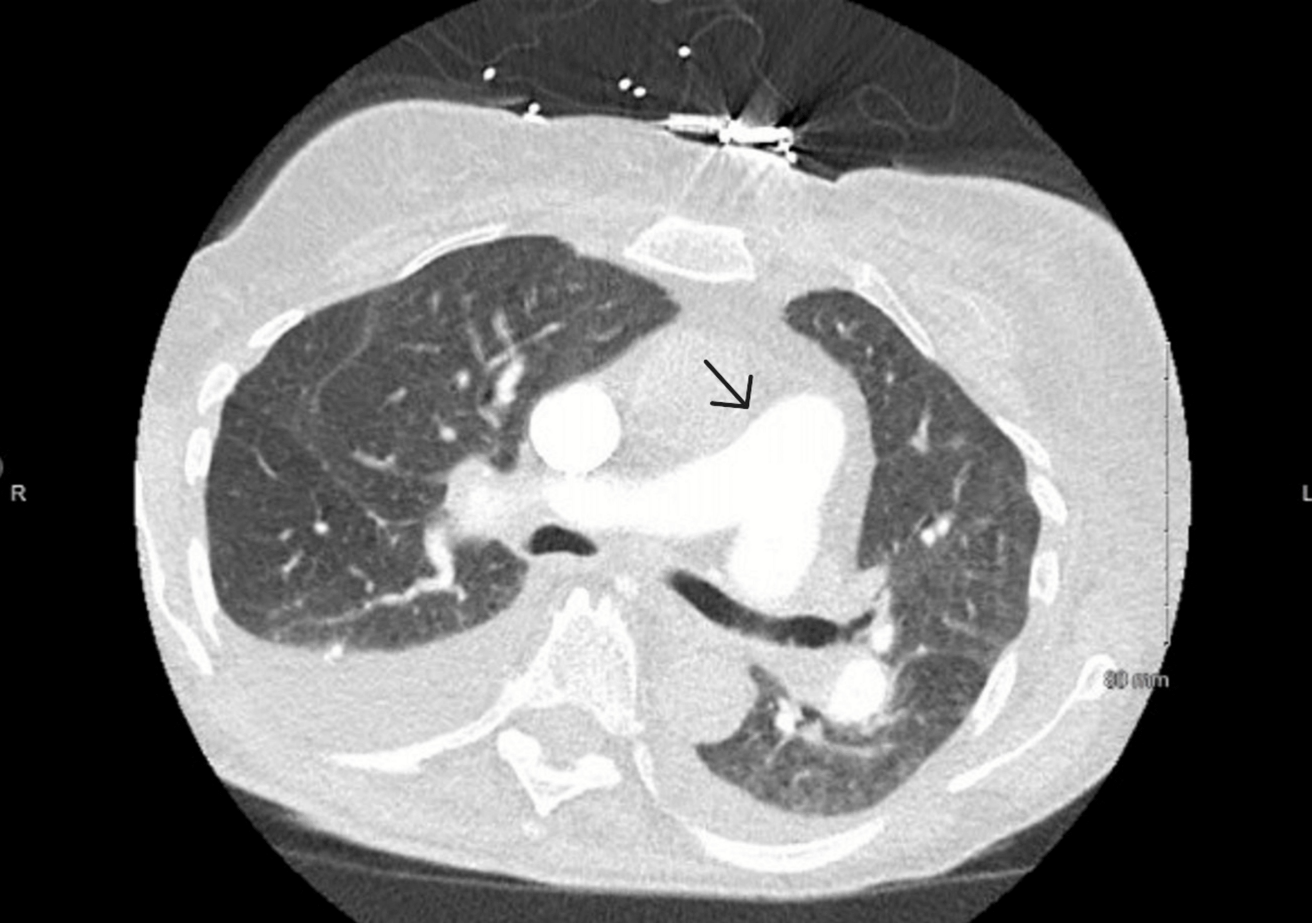
CT angiogram of the chest showed marked right heart and pulmonary artery enlargement (see arrow)

Electrocardiogram (ECG) exhibited sinus rhythm with occasional PVCs, possible left atrial enlargement, QTc 450 ms, and a persistent right bundle branch block. Bilateral leg radiographs showed soft tissue swelling but no gas or osteomyelitis. A CT angiogram of the chest excluded pulmonary embolism; findings included marked right heart and pulmonary artery enlargement, small bilateral pleural effusions, emphysematous changes, and apical nodular scarring consistent with prior sarcoidosis (Figure 2).

Due to suspicion of right heart failure and cellulitis on her legs, she was started on IV diuretics and broad-spectrum antibiotics. Her renal function, liver enzymes, and leukocytosis improved; blood cultures grew methicillin-sensitive *Staphylococcus auerus* (MSSA), confirming the leg cellulitis as the source of sepsis, and therapy was narrowed to IV cefazolin. A noncontrast CT abdomen/pelvis showed mild ascites and a 9 cm pelvic fluid collection anterior to the uterus. CT-guided aspiration yielded 480 mL of serous fluid and the gram stain, culture, and cytology of ascitic fluid were negative. A repeat TTE showed preserved left ventricular systolic function (EF 65-70%), but severe right ventricular (RV) dilation, mild RV systolic dysfunction, severe tricuspid regurgitation, right atrial enlargement, and estimated RV systolic pressure of 67 mmHg. No vegetations were seen. A right heart catheterization was performed, which showed pulmonary capillary wedge pressure (PCWP) 9 mmHg, mean pulmonary artery pressure (mPAP) 33 mmHg (46/26), and pulmonary vascular resistance (PVR) 8.6 Wood units, confirming precapillary PH. Considering her sarcoidosis and NF1, her PH was considered multifactorial, with predominant small-vessel pulmonary vascular remodeling. She was started on sildenafil, with symptomatic improvement. After resolution of MSSA, he was discharged on oral furosemide 40 mg daily, sildenafil 20 mg three times a day, and aspirin 81 mg daily. 

At eight-week follow-up, she remained stable; sildenafil was increased to 40 mg three times a day, and macitentan 10 mg daily was added. Her exertional dyspnea improved. Further workup was planned, including repeat PFTs with diffusing capacity for carbon monoxide (DLCO), six-minute walk test, a sleep study, and surveillance imaging. On follow up, she was prescribed selexipag, an oral prostacyclin receptor agonist, in a dose of 200 mcg twice daily to control PH. Sotatercept, a novel fusion protein composed of extracellular domain of the human activin receptor Type IIA fused to the Fc domain of human IgG1, which acts as a ligand trap for members of transforming growth factor (TGF)-β superfamily, thereby controlling PH, was offered, but she declined as she was uncomfortable administering subcutaneous injections to herself [10]. 

## Discussion

We present an interesting case of severe PH in a postmenopausal woman with NF1 and sarcoidosis. She was diagnosed with breast cancer 30 years ago, which was treated with a left mastectomy followed by chemotherapy, and she has remained in remission since then. While her remote history of chemotherapy for breast cancer may raise concern for pulmonary vascular disease, it is a less plausible etiology in this case. 

She received chemotherapy for breast cancer in 1995, likely including cyclophosphamide and anthracyclines [11,12]. Cyclophosphamide has been implicated in pulmonary veno-occlusive disease (PVOD), a rare cause of precapillary PH characterized by post-capillary obstruction, profound hypoxemia, and markedly reduced DLCO [13]. However, PVOD typically manifests within weeks to months of exposure, not decades later [14]. Moreover, this patient had normal PFTs in 2014 and was not hypoxemic on presentation, making a PVOD-like syndrome or chemotherapy-induced PH unlikely. 

**Figure 3:**
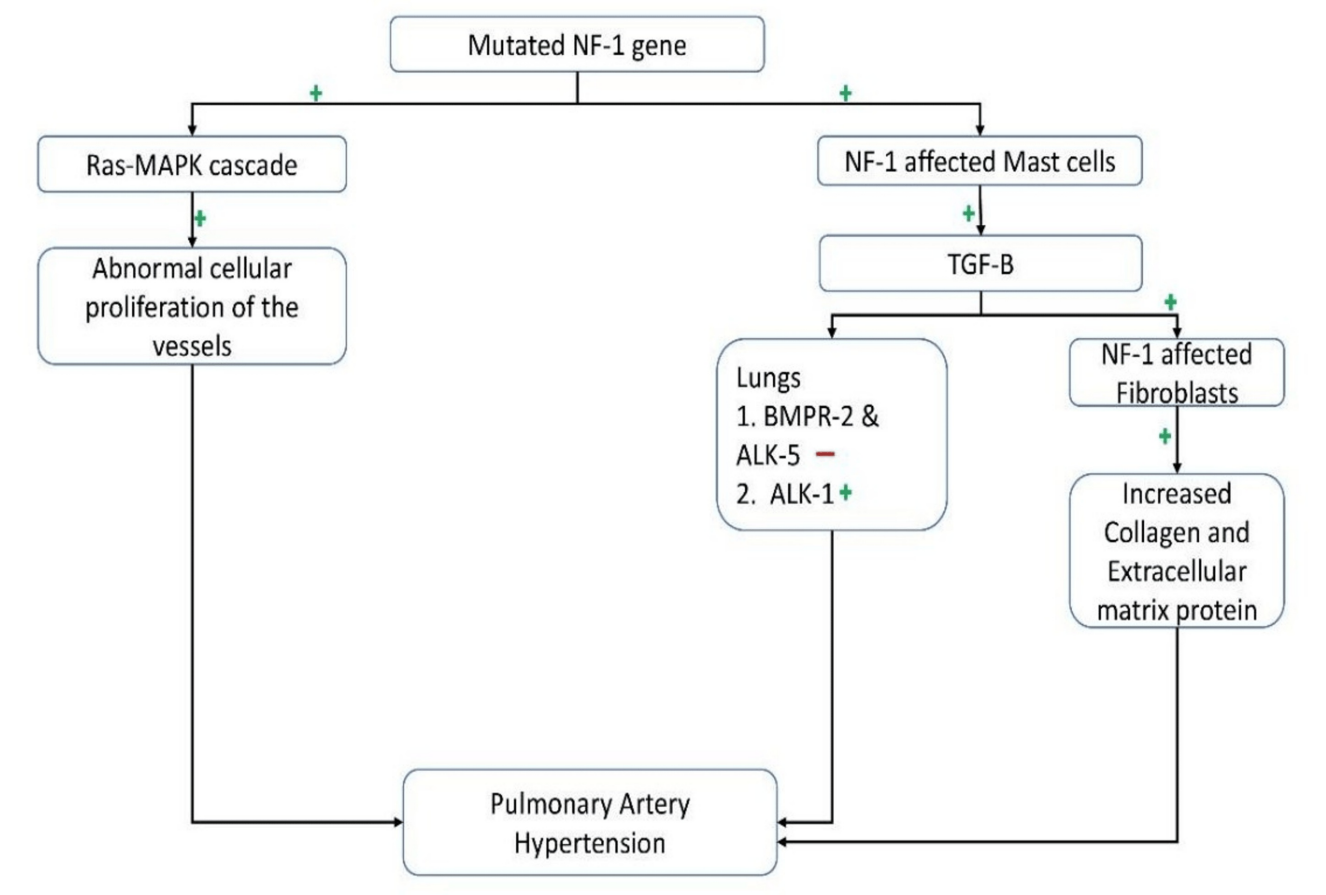
Mechanisms behind NF1 causing precapillary PH See [4]. NF1: Neurofibromatosis Type 1; PH: Pulmonary hypertensio

The most probable contributor to her PH is NF1-associated PAH, a rare but increasingly recognized entity. The precise mechanisms are incompletely understood, but several molecular pathways have been proposed. NF1 results from mutations in the NF1 gene, which encodes neurofibromin, a tumor suppressor that negatively regulates the Ras-MAPK signaling pathway. Loss of neurofibromin function leads to hyperactivation of Ras-dependent signaling, promoting vascular smooth muscle cell proliferation, and pulmonary vascular remodeling [4]. In addition, mast cells in NF1 have been shown to secrete TGF-β, which activates fibroblasts to increase extracellular matrix deposition and fibrosis [4]. This cascade may interact with signaling via bone morphogenetic protein receptor type 2 (BMPR2) and activin receptor-like kinase pathways-both of which are implicated in the pathobiology of idiopathic and heritable PAH (Figure 3) [4].

In a small cohort study, PAH-targeted therapies-including endothelin receptor antagonists, phosphodiesterase-5 (PDE5) inhibitors, and prostacyclin analogs-have been associated with improvements in mPAP, PVR, and NYHA functional class in NF1-associated PAH [3]. However, some patients may develop worsening hypoxemia or demonstrate limited response over time, underscoring the need for close monitoring. Interestingly, a recent case series reported that sorafenib, a multikinase inhibitor targeting Raf-1, produced meaningful improvements in mPAP and PVR over three months, suggesting that tyrosine kinase inhibitors (TKIs) targeting Ras-MAPK signaling could represent a future therapeutic strategy in this population [15]. 

Our patient responded well to sildenafil, a PDE5 inhibitor, without evidence of hypoxemia or worsening gas exchange, which supports a vasodilator-responsive phenotype. 

**Figure 4:**
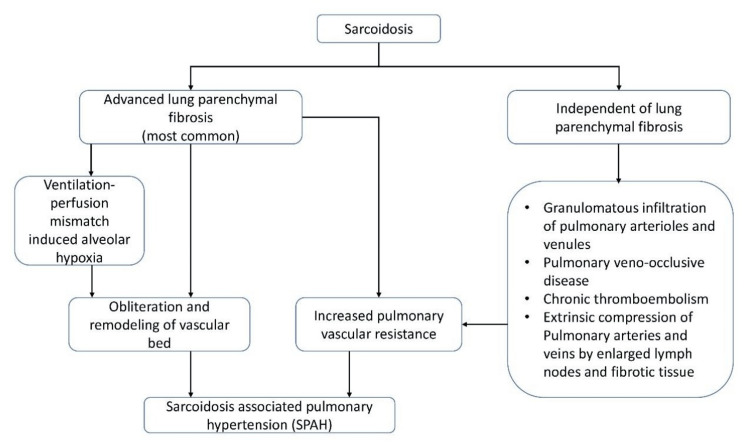
Mechanisms behind sarcoidosis causing precapillary PH See [7]. PH: Pulmonary hypertension

SAPH is a well-described complication, with a reported prevalence ranging 3-20% among sarcoidosis patients and up to 74% in those with advanced pulmonary involvement [7]. SAPH is pathophysiologically heterogeneous, with multiple proposed mechanisms (Figure 4), including pulmonary vasculopathy from granulomatous inflammation of vessels, destruction of the pulmonary capillary bed due to parenchymal fibrosis, chronic alveolar hypoxia leading to hypoxic vasoconstriction, extrinsic vascular compression from fibrosing mediastinitis or lymphadenopathy and left heart dysfunction due to cardiac sarcoidosis [7].

Most cases of SAPH exhibit a precapillary hemodynamic profile, as seen in our patient, although postcapillary or mixed forms may occur, particularly when cardiac sarcoidosis is involved. In this case, the patient had a normal PCWP and severe RV dilation, without echocardiographic features of left-sided heart failure, supporting a precapillary process. While our patient’s CT chest demonstrated apical nodular scarring, there was no extensive fibrosis or traction bronchiectasis, and her gas exchange remained preserved. These findings suggest that her PH was not primarily driven by parenchymal fibrosis due to sarcoidosis. PAH-targeted vasodilator therapy has shown benefit in select group of patients with SAPH, especially when vascular remodeling is the dominant process [16]. Our patient’s clinical improvement on sildenafil and subsequent initiation of macitentan, an endothelin receptor antagonist, supports this approach. However, vasodilator therapy may worsen ventilation-perfusion mismatch in patients with significant fibrotic lung disease and must be used cautiously [17]. Importantly, if SAPH is driven by active granulomatous vasculitis or extrinsic vascular compression, immunosuppressive therapy, such as corticosteroids or steroid-sparing agents (e.g., methotrexate, azathioprine, or tumor necrosis factor (TNF)-α inhibitors), may be indicated [17,18]. In this case, her cutaneous and ocular sarcoidosis were inactive, and there was no radiographic or biomarker evidence of systemic inflammatory activity, making immunosuppression unnecessary at this stage. Further evaluation with positron emission tomography (PET)-CT or soluble IL-2 receptor levels may be warranted to exclude occult inflammatory activity in follow-up. 

NF1-associated PH was most likely in this case as her sarcoidosis was not active and apart from some old apical fibrosis, she did not have significant lung fibrosis, traction bronchiectasis, multiple lung nodules, cysts, cavities, reticular changes or enlarged lymphadenopathy. Also, she was not requiring any steroids or immunosuppressants for her sarcoidosis. There was no evidence of cardiac sarcoidosis either.

## Conclusions

This case highlights the diagnostic complexity of multifactorial precapillary PH in a patient with NF1 and sarcoidosis, both of which are recognized but rare contributors to PH. Early hemodynamic evaluation and targeted therapy with PAH-directed vasodilators led to meaningful clinical improvement. Long-term outcomes in NF1-associated PAH remain poor in reported cohorts, underscoring the importance of close monitoring, consideration of alternative therapeutic targets (e.g., Ras-MAPK inhibition), and early referral to a specialized PH center. 
